# Disappearance of Plaque Following Treatment with Antioxidants in Peyronie’s Disease Patients—A Report of 3 Cases

**DOI:** 10.3390/clinpract12060105

**Published:** 2022-12-09

**Authors:** Gianni Paulis, Giovanni De Giorgio

**Affiliations:** 1 Peyronie’s Care Center, Department of Urology and Andrology, Castelfidardo Clinical Analysis Center, 00185 Rome, Italy; 2 Section of Ultrasound Diagnostics, Department of Urology and Andrology, Castelfidardo Clinical Analysis Center, 00185 Rome, Italy

**Keywords:** Peyronie’s disease, oxidative stress, antioxidants, pentoxifylline

## Abstract

Peyronie’s disease (PD) is a fibrotic disorder of the penile tunica albuginea. To date, only a few cases of recovery from PD following medical treatment have been reported in the literature. In this article, we describe three new cases of PD where patients achieved complete resorption of plaque following multimodal antioxidant treatment. In all three cases, treatment included the following antioxidants: bilberry, propolis, ginkgo biloba, silymarin, and vitamin E. Only in case nos. 1 and 2 did we also use the following antioxidant substances: L-carnitine, coenzyme Q10, and Boswellia. In all three cases, we also used a local therapy with diclofenac gel. Only in case no. 2 did we also use periodic perilesional injections with pentoxifylline. Although the sample of cases presented here was small, these patients incontrovertibly experienced complete plaque disappearance and recovery (in one case, only after a short course of treatment). Therefore, it is our conviction that urologists may find our experiences of considerable interest in their clinical practices.

## 1. Introduction

Peyronie’s disease (PD) affects adult men and is characterized by chronic inflammation of the tunica albuginea of the penile corpora cavernosa. Its hallmark is the formation of fibrous plaque, which may even undergo calcification and result in the deformation of the penis. PD symptoms are as follows: penile deformity (shortening, curvature, divots, hourglass appearance, etc.), pain in the penis (20–70%), erectile dysfunction (approximately 30%), and anxiety/depression (48%) [1,2,3].

PD prevalence ranges from 3.2 to 13% [4,5,6]. Although its etiopathogenesis has yet to be fully explained, PD is known to affect males who have a genetic susceptibility, and it seems to be triggered by trauma, which may be mild or severe and is variously associated with risk factors, such as diabetes, chronic prostatitis, autoimmune disorders, hypertension, etc. [7,8,9,10].

Disease onset occurs as blood and fibrin accumulate at the site of the trauma, inducing the recruitment of inflammatory cells and excessive production of fibrogenic factors and reactive oxygen species [11,12]. Oxidative stress, in particular, was recently found to be crucial to plaque formation and the evolution of PD itself [13,14].

Surgical intervention involves the treatment of choice when PD has reached its stable phase and in cases presenting with severe erectile dysfunction and/or severe penile curvature preventing complete sexual intercourse, while medical therapy is indicated instead when PD is in its initial, inflammatory phase. Medical therapy comprises oral treatment with Potaba, colchicine, tamoxifen, vitamin E, phosphodiesterase-5 inhibitors, antioxidant substances, penile injections with anti-fibrotic and anti-inflammatory substances, antioxidants (pentoxifylline, verapamil, corticosteroids, collagenase, interferon, etc.), as well as physical treatments (extracorporeal shock wave therapy, iontophoresis, vacuum, and penile traction devices) [15,16,17].

We have already published articles reporting on the full recovery of patients following antioxidant treatment.

There was no photographic documentation in previous case reports/articles because the patients did not authorize the publication of photographs of their penis.

We present here three new cases of PD where patients experienced complete plaque disappearance following multimodal antioxidant therapy at our PD Care Center.

For two of these cases, photographs before and after treatment are included. Furthermore, unlike the previous articles, in case no. 3, we obtained complete regression of the disease in just four months.

## 2. Case Presentations

### 2.1. Case 1

A 31-year-old non-smoking Caucasian man suffering from chronic prostatitis and an associated anxious–depressive state presented to our clinic in March 2019. He complained of painful erections and a penile bend that commenced approximately nine months before. The patient was asked to rate his pain using a visual analog score (VAS); his VAS was 8 (range 0–10). He was then asked to complete the International Index of Erectile Function (IIEF) questionnaire.

**Figure 1:**
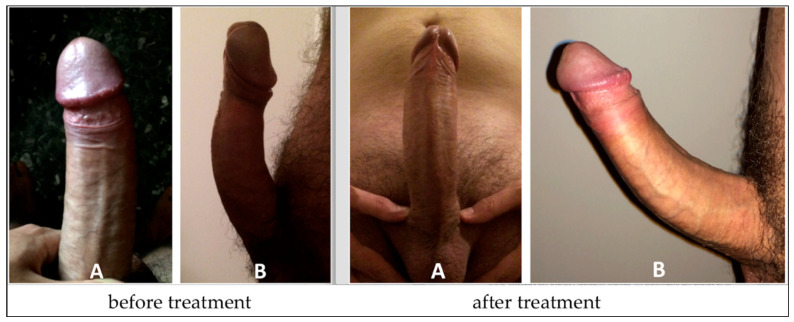
Case 1. Anterior (**A**) and lateral (**B**) photographic poses (before and after treatment).

We considered questions 1, 2, 3, 4, 5, and 15 regarding erectile function (normal range 26–30). The patient’s score was 26. The patient’s penis presented both a 10-degree ventral curvature and a lateral curvature with a 15-degree bend to the left (Figure 1, before treatment).

Upon physical examination, a fibrous nodule measuring approximately 10 mm was palpable at the distal third of the penis.

A penile Doppler ultrasound scan was performed after erection was induced by injection of Alprostadil 10 mcg. The formula of the ellipsoid (volume = 0.524 × length × width × thickness) was used to take three-dimensional measurements of the plaque volume [18,19].

**Figure 2:**
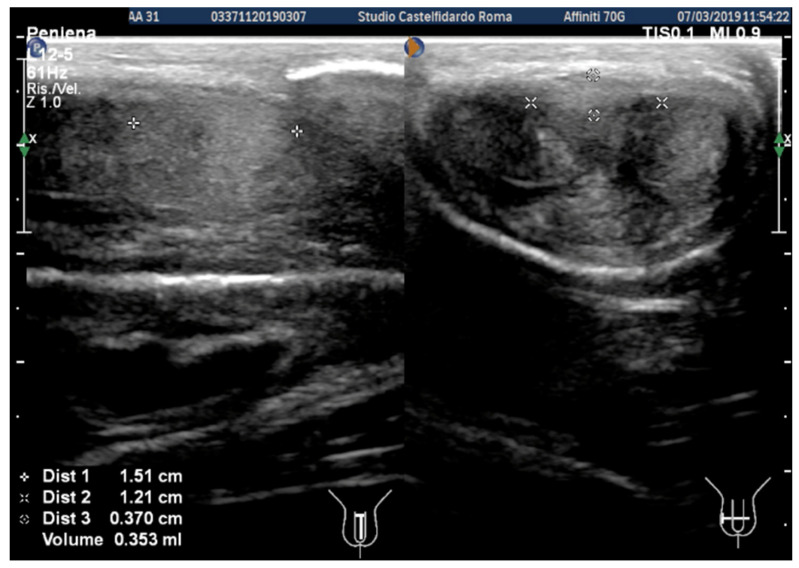
Ultrasonography of the penis before treatment (longitudinal and transverse views).

Cavernous artery flow and end-diastolic velocity were normal. Bilaterally, the peak systolic velocity was 90 cm/s and the end-diastolic velocity was 0 cm/s. The plaque, which was situated at the distal third of the penile shaft, was isohypoechoic, measuring 15.1 × 12.1 × 3.7 mm (volume = 353 mm^3^) (Figure 2, Table 1).

When gathering the patient’s informed consent, the patient was forewarned that the treatment would be lengthy, considering the disease’s chronicity. Fearing pain, the patient refused to receive penile infiltrations. The patient began treatment in March 2019 after granting his informed consent. Treatment comprised of the following: oral propolis 600 mg + bilberry 160 mg + silymarin 400 mg + ginkgo biloba 250 mg + L-carnitine 1000 mg + coenzyme Q10 100 mg + Boswellia 200 mg + vitamin E 30 mg/daily; diclofenac gel 4%/2× per day for 12 months.

**Figure 3:**
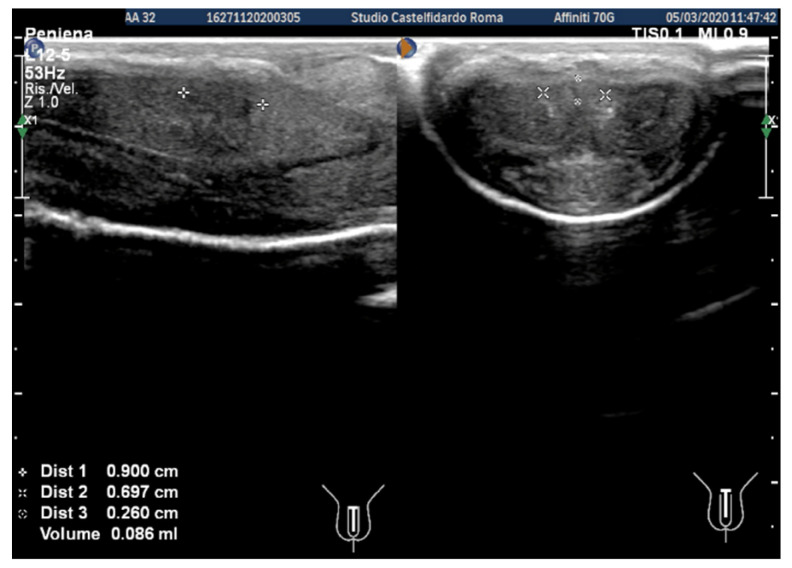
Ultrasonography of the penis after the first treatment cycle (longitudinal and transverse views).

The patient then underwent his first follow-up. He filled out the IIEF questionnaire, and his score (26) had not varied. We then observed a reduction to 5 degrees in the lateral left penile curvature angle, and we no longer observed any ventral penile curvature (previously present with a 10-degree angle). Erections were no longer painful. Dimensions on the ultrasound were as follows: 9.0 × 6.97 × 2.6 mm (volume = 86 mm^3^) (Figure 3).

The plaque volume was, therefore, 75.6% smaller than before treatment. In view of the good results achieved after the first cycle, a second identical 12-month treatment cycle was agreed on with the patient.

**Figure 4:**
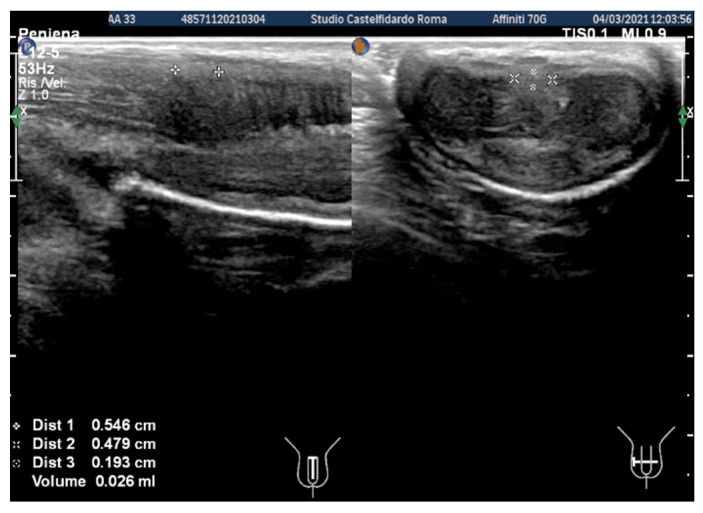
Ultrasonography of the penis after the second treatment cycle (longitudinal and transverse views).

After the second treatment cycle, at the 24-month follow-up, the IIEF score had risen to 27, and a lateral left penile bend of 2–3 degrees could be observed. The ultrasound dimensions were 5.46 × 4.79 × 1.93 mm (volume = 26 mm^3^) (Figure 4).

The plaque’s volume had, therefore, decreased by 92.6%. In view of the excellent results after the second cycle, the decision was made to continue treatment, with no changes, for only six more months.

**Figure 5:**
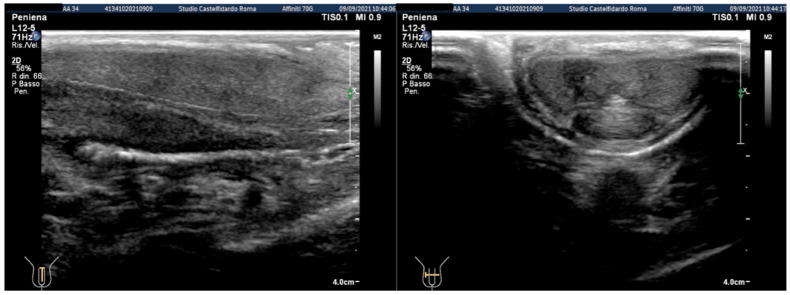
Ultrasonography of the penis after the third treatment cycle (longitudinal and transverse views).

At follow-up, after three full treatment cycles corresponding to an overall duration of therapy of two years and six months, the patient had the same IIEF score. Penile palpation did not detect any nodule and the two original curvatures were observed to have disappeared (Figure 1, after treatment—see above). Plaque was no longer visible on the ultrasound (Figure 5).

The same physician performed the initial ultrasound scan as well as all follow-up scans, always employing the same Philips Affinity 70 G device (Philips, Seattle, WA, USA).

Antioxidant treatment was consequently suspended. Following treatment, the patient reported no side effects.

### 2.2. Case 2

A 52-year-old Caucasian man, non-smoker, with Dupuytren’s disease and Ledderhose disease, who had a 5-degree congenital dorsal penile curvature before PD onset, presented to our clinic in February 2015; the patient did not complain of any pain in the penis. At the time of our observation, the penis presented both a dorsal curvature, with a 20-degree angle, and a lateral left curvature of 20 degrees. The patient was, therefore, asked to fill in the IIEF questionnaire on erectile function, which yielded a score of 26. Upon physical examination, a fibrous nodule, approximately 25 mm long, was palpable at the middle and distal third of the penile shaft. After inducing erection with the injection of Alprostadil 10 mcg, a penile Doppler scan was performed.

Cavernous artery flow and end-diastolic velocity were normal: peak systolic velocity = 70 cm/s on the right and 74 cm/s on the left; end-diastolic velocity = 0 cm/s on both sides.

**Figure 6:**
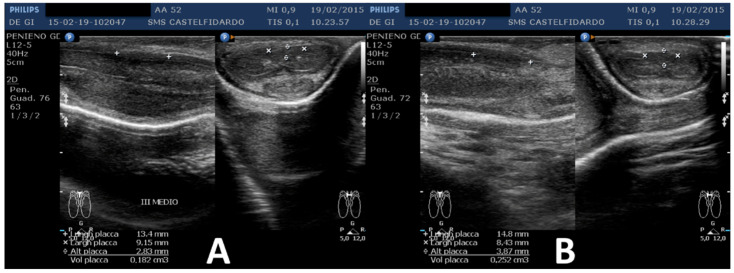
Ultrasonography of the penis and plaque measurement before treatment (longitudinal and transverse views). (**A**) First plaque, (**B**) second plaque.

The ultrasound scan showed multifocal disease consisting of two penile plaques with isohypoechoic appearance. The first plaque, located at the middle third of the penile shaft, measured 13.4 × 9.15 × 2.83 mm (volume = 182 mm^3^); the second plaque, located at the distal third, measured 14.8 × 8.43 × 3.87 mm (volume = 252 mm^3^) (total volume of the two plaques = 434 mm^3^) (Figure 6, Table 1).

When gathering the patient’s informed consent, the patient was forewarned that the treatment would be lengthy, considering the disease’s chronicity. The patient did not authorize the publication, however anonymous, of photographs of his penis. In March 2015, the patient provided his informed consent and began the following combined treatment: oral propolis 600 mg + bilberry 160 mg + silymarin 400 mg + ginkgo biloba 250 mg + L-carnitine 1000 mg + coenzyme Q10 100 mg + Boswellia 200 mg + vitamin E 30 mg/daily; topical diclofenac gel 4%/2× per day; pentoxifylline 100 mg periplaque injection (with a 30G needle) every two weeks for six months. In order to minimize trauma to the penile tissues, penile injections with pentoxifylline were performed in a perilesional fashion (and not inside the plaque). For the same reason, a very thin needle (30 G) was used.

Having ended his first therapy cycle, the patient was followed up with the same examination process as previously. His IIEF score had risen to 27. A dorsal penile curvature could still be observed, but its angle was reduced to 15 degrees; we no longer observed any lateral penile curvature.

**Figure 7:**
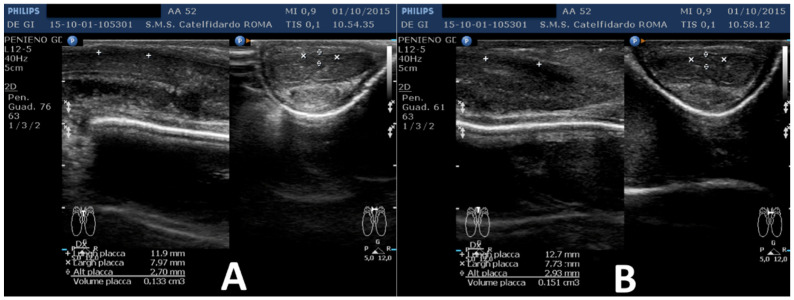
Ultrasonography of the penis after the first treatment cycle (longitudinal and transverse views). (**A**) First plaque. (**B**) Second plaque.

Plaque dimensions on the ultrasound were as follows: the first plaque, located at the middle third of the penile shaft, measured 11.9 × 7.97 × 2.7 mm (volume = 133 mm^3^); the second plaque, located at the distal third, measured 12.7 × 7.73 × 2.93 mm (volume = 151 mm^3^) (total volume of the two plaques = 284 mm^3^) (Figure 7).

The total volume of the two plaques was 34.5% smaller compared to the initial total volume. Since the first cycle of the multimodal therapy had yielded a good response, the decision was made to continue the same oral and topical treatments, with the same agents and doses, for a further 12 months, while reducing pentoxifylline’s 100 mg injection frequency to one penile injection every month for 12 months.

The patient then underwent follow-up with the IIEF questionnaire, physical exam, and penile Doppler ultrasound after the second cycle of treatment. His IIEF score was 27. We observed that the dorsal penile curvature was unchanged (15 degrees); we did not observe any lateral penile curvature.

**Figure 8:**
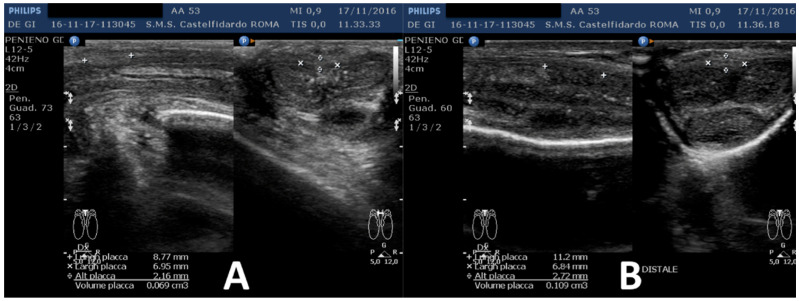
Ultrasonography of the penis after the second treatment cycle. (**A**) First plaque, (**B**) second plaque.

The ultrasound dimensions were as follows: the first plaque, located at the middle third of the penile shaft, measured 8.77 × 6.95 × 2.16 mm (volume = 69 mm^3^); the second plaque, located at the distal third, measured 11.2 × 6.84 × 2.72 mm (volume = 109 mm^3^) (total volume of the two plaques = 178 mm^3^) (Figure 8).

The total size of the two plaques was 58.9% smaller compared to the original dimensions. In consideration of the good response achieved by the second treatment cycle, the decision was made to continue the same treatment for a third cycle, with the same agents and doses, while reducing pentoxifylline’s 100 mg injection frequency to one penile periplaque injection every other month.

After completion of the third treatment cycle, the patient underwent a follow-up consisting of a physical exam and penile Doppler ultrasound. At the follow-up, the patient’s IIEF score was 27. We observed that the dorsal penile curvature reduced to 10 degrees.

**Figure 9:**
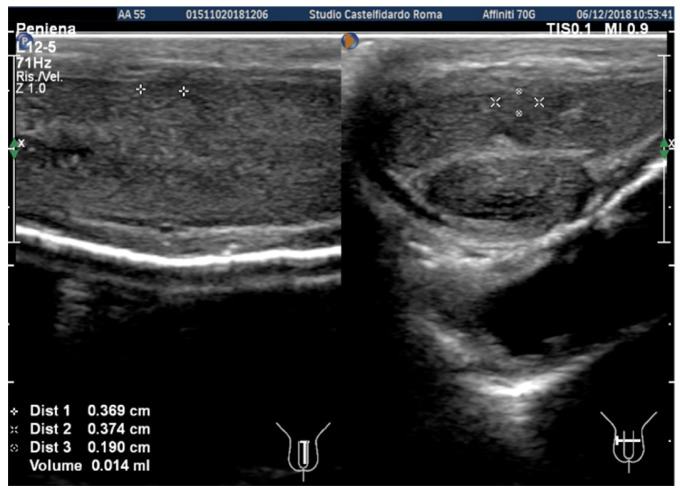
Ultrasonography of the penis after the third treatment cycle (longitudinal and transverse views).

The penile ultrasonography detected the presence of only one plaque (middle third), whereas the distal plaque was no longer detectable. Plaque dimensions on the ultrasound were 3.69 × 3.74 × 1.9 mm (volume = 14 mm^3^) (Figure 9).

Since the previous examinations, the ultrasound device (Philips HD 15, Seattle, WA, USA) in our clinic had been replaced with a new device (Philips Affinity 70 G, Seattle, WA, USA), which was used in the latter and subsequent follow-ups. The same doctor performed all sonographic examinations.

Upon completion of three treatment cycles, the total plaque volume was 96.7% smaller compared to the initial total volume of the two plaques. In view of the excellent response to the third multimodal treatment cycle, we elected to cease the periplaque injections with pentoxifylline and extend the oral and topical treatments (unchanged for an additional six months).

**Figure 10:**
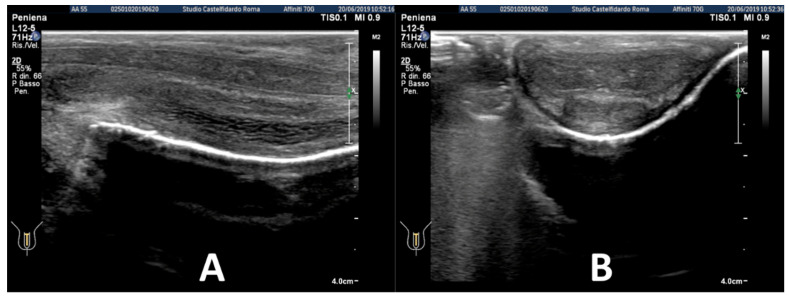
Ultrasonography of the penis after the fourth treatment cycle. (**A**) longitudinal view, (**B**) transverse view.

After four full treatment cycles, for an overall course of multimodal therapy lasting approximately four years and three months, the patient underwent follow-up with the IIEF questionnaire, physical exam, and penile Doppler ultrasound. No change was observed in the IIEF score, which was again 27. The angle of the dorsal penile curvature decreased to 5 degrees, a measure comparable to the condition of the patient before PD onset (congenital curvature of the penis). No nodule could be detected on palpation. Plaque was no longer visible on the ultrasonography (Figure 10, Table 1). Consequently, our antioxidant treatment was suspended.

Following treatment, the patient reported no side effects. The same physician performed all ultrasonography studies.

### 2.3. Case 3

**Figure 11:**
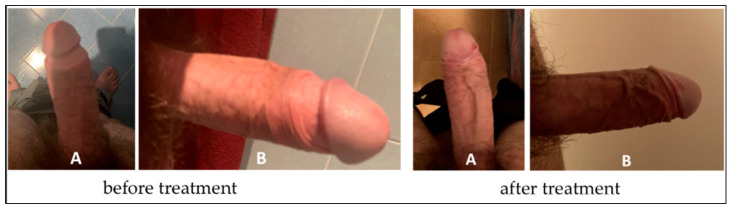
Case 3. Anterior (**A**) and lateral (**B**) photographic poses (before and after treatment).

A 30-year-old Caucasian man, non-smoker, with left varicocele, presented to our clinic in September 2014 complaining of pain in the penis with erections, with onset approximately one week earlier. The patient reported penile sexual trauma four weeks before. At the time of our observation, no penile deformity was present. The VAS score was 5. The IIEF score was 26. No nodules were found on penile palpation. Following the physical examination, a penile Doppler ultrasound was performed. After the erection was induced with 10 mcg of the intracavernous injection of Alprostadil, the erect penis showed no deformation (Figure 11, before treatment).

End-diastolic velocity and the cavernous artery flow were normal; the end-diastolic velocity = 0 cm/s (bilaterally); peak systolic velocity = 84.9 cm/s on the left and 77.3 cm/s on the right.

**Figure 12:**
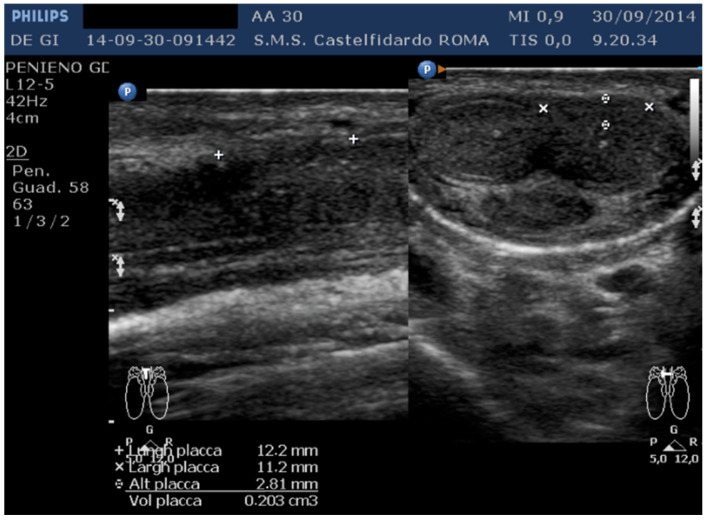
Ultrasonography of the penis and measurement of plaque before treatment (longitudinal and transverse views).

The penile plaque was situated at the proximal third, the plaque was isohypoechoic, and measurements were 12.2 × 11.2 × 2.81 mm (volume = 203 mm^3^) (Figure 12, Table 1).

When gathering the patient’s informed consent, the patient was forewarned that the treatment would be lengthy, considering the chronicity of the disease.

After receiving the patient’s informed consent, on 30 September 2014, we began the following combination therapy with oral antioxidants and topical NSAIDs: bilberry 160 mg + propolis 600 mg + silymarin 400 mg + vitamin E 800 IU + ginkgo biloba 250 mg/per day + diclofenac gel (4%) twice daily for six months.

Respecting the patient’s decision (the patient was very anxious), follow-up was performed only four months after the beginning of treatment. No change was observed in the IIEF score, which was still 26. Erections were no longer painful. No penile deformation was observed (Figure 11, after treatment—see above).

**Figure 13:**
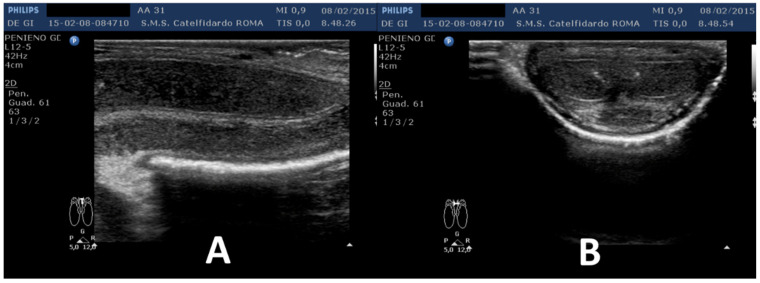
Ultrasonography of the penis after the first treatment cycle. (**A**) Longitudinal view, (**B**) transverse view.

Plaque was no longer visible on the ultrasound (Figure 13, Table 1).

Accordingly, after approximately four months of multimodal antioxidants, treatment was suspended. Following treatment, the patient reported no side effects.

The same physician performed the initial ultrasound scan as well as all follow-up scans, always using the same Philips HD 15 device (Philips, Seattle, WA, USA).

## 3. Discussion

Although antioxidants are not included in current AUA and EAU guidelines for PD treatment, in the literature, there are three randomized studies describing the use of antioxidant substances in patients suffering from PD, as well as a number of controlled studies where antioxidants were used in association, with excellent benefits (plaque reduction and an improvement in penile deformity) [20,21,22,23,24,25,26,27].

The European Association of Urology (EAU) guidelines and American Urological Association (AUA) guidelines strongly recommend infiltrative therapy with interferon alpha-2b or with collagenase clostridium histolyticum CCH [26,27].

We have never used interferon alpha-2b because of its high cost and the high possibility of side effects, such as diarrhea, dizziness, flu-like symptoms, nausea, fatigue, vomiting, etc. We have also never used CCH in treatment because its indications are Peyronie’s disease in the “stabilization phase”, and our PD patients are all in the active phase of the disease. It should be added that, in Italy, the drug Xiapex (CCH) (from 1 January 2020) was withdrawn from the market by the Italian Medicines Agency (AIFA).

In recent years, we adopted the combination of antioxidants presented in this case report article. Our multimodal (or combined) treatment consisted of administering several therapeutic agents in various ways (by ingestion, injection, or as topical medication) and sometimes in association with iontophoresis. Multimodal therapy further improved outcomes compared with the results that may be achieved while employing a single active substance or therapy.

Initially, many years ago, we only used propolis, bilberry, and then also pentoxifylline, but in this way, we only achieved a partial response. Therefore, we decided to progressively increase the number of substances, recently obtaining better results up to the complete regression of the plaque. We decided on the dosages for each substance while trying to respect the therapeutic dosage to avoid side effects.

In this regard, we consulted other studies that had used some of the same substances. We have not used pentoxifylline orally for several years because side effects have previously occurred (nausea, vomiting, dyspepsia, meteorism, hot flashes, and headache) (see our study) [21]. We, therefore, decided to use pentoxifylline only for local administration (peri-lesional penile injection) and only in the case of penile plaques with greater extension. Each antioxidant substance that we use in the treatment has specific antioxidant, antifibrotic, and anti-inflammatory properties, and each of them is therefore able to interfere with the chronic inflammation and “oxidative stress” present in Peyronie’s disease [14]. These substances not only antagonize “oxidative stress” but block the activity of numerous cytokines that are active in the disease and responsible for its progression (transforming growth factor beta, platelet-derived growth factor, basic-fibrotic growth factor, interleukin-1, etc.) [14].

Recently, we published three case report articles, where we presented eight patients (in total) with PD whose plaque disappeared completely after multimodal antioxidant treatment [28,29,30]. There was no photographic documentation in these case reports. Instead, in the present article, for two of these cases, photographs before and after treatment are included.

Prior to our articles, the disappearance of PD plaque had already been described in an experimental study on rats [31].

In the first two cases reported here, the period of time needed for our treatment was lengthy; the duration of treatment is due to the fact that, as PD is a “chronic” inflammatory disease, it takes time to achieve full plaque resorption. The necessary length of treatment is also linked to the size of the diseased area.

It is likely that the excellent result achieved in the third case, with a markedly shorter treatment time compared with the other two cases, can be mainly ascribed to the fact that diagnosis was made extremely early in the disease (four weeks after trauma). In this case, it was possible to have the patient undergo a shorter cycle of treatment, which did not require periodic penile injections, as the plaque was limited in size, clearly because the disease was in an extremely early phase.

In the last case presented, the ultrasound examination (performed with the latest generation machine) clearly detected a plaque with an ultrasound appearance typical of the first phase of the disease, i.e., hypoechoic. Furthermore, after four months of treatment, the plaque was no longer visible in the ultrasound examination. The deformation was absent because the plaque was newly formed; therefore, it was not yet fibrotic and was unable to cause a deformation in the form of curvature. We know from the literature that curvature is not always present during PD (present in 87–94% of cases) [2,3,14].

Although the sample of cases presented here is small, we believe all urologists may find our experience of great interest for their clinical practice.

In the period 2014–2018, we treated 45 PD patients with similar multimodal treatment. Of these patients, 22 successfully completed the treatment until complete plaque regression (after a variable period between 28 and 53 months).

In total, 18 of these patients also underwent periodic penile injections with pentoxifylline. The remaining 23 patients discontinued treatment after approximately 13–24 months despite the reduction in plaque volume and the improvement in the curve over time. These 23 patients felt that the treatment was too long. We believe that when the plaque volume is large, it takes some years to achieve complete regression of the disease (chronic inflammation).

In our opinion, the success achieved by our therapy rests in great part on the properties of the antioxidants we employed [12,13,14].

The antioxidant substances we used also inhibit the NF-kB factor, a transcription factor for the production of pro-inflammatory and pro-fibrotic cytokines [32,33,34,35,36,37,38]. In our topical therapy, we also used diclofenac, which, besides its anti-inflammatory and antioxidant properties, has been proven to deeply penetrate the tissues [39,40].

When our treatment plan includes injection therapy, we gradually increase the time interval between each pentoxifylline injection over the course of treatment. The reason behind this is that even simple peri-lesional injections implicate trauma, be it ever so mild, and as trauma is beyond any doubt the trigger for the onset of PD, it should be avoided as much as possible [7,8,9]. Therefore, whenever any disease regression is observed at follow-up, we opt to space out the injections to minimize the possibility of any new trauma.

Whereas ultrasonography in the literature is generally deemed inadequate for acceptable measurements of plaque, in our experience, plaque dimensions can be accurately obtained if an experienced physician performs the scans using a highly sensitive, cutting-edge ultrasound machine [26,41,42]. Therefore, we attribute our good outcomes on the one hand to the antioxidants used in treating patients, and on the other to the nature of our ultrasound assessments, which enabled us to provide an accurate diagnosis of the area affected by the disease (plaque) and keep track of the disease’s evolution at scheduled follow-ups.

The limitations of this study are the limited number of patients and the relative absence of a control group.

## 4. Conclusions

Although the sample of cases presented here is small, these patients incontrovertibly experienced the complete disappearance of plaque in the area affected by the disease, in one case after only a brief course of treatment.

We attribute our success fundamentally to the following three factors: the choice of antioxidants used; the performance of ultrasonography with an extremely sensitive ultrasound machine by which we obtained precise plaque measurements; and our entrusting the ultrasound scan to a physician with extensive experience with PD cases.

Therefore, it is our conviction that specialists may find our report, however limited, of great interest for their urology and andrology practice.

Still, there is a need for randomized controlled studies enrolling a larger number of patients in order to prove the effectiveness of this multimodal antioxidant treatment for patients suffering from PD.

## Figures and Tables

**Table 1:** Case summaries of three patients with Peyronie’s disease treated with combined multimodal therapy.

No.	Patient Age	Associated Disease	Penile Plaque Site	Ultrasound Measurements (Length × Width × Thickness) and Plaque Volume before (A) and after Treatment (B)	Type of Deformity Initial (A) and after Treatment (B)	Pain Score/VAS Scale (1–10)	IIEF Score before and after Treatment	Total Duration of Treatment until Plaque Regression	Complete Combined Multimodal Treatment
1	31 years	Chronic prostatitis and associated anxious and depressive state.	Distal third	(A) 15.1 × 12.1 × 3.7 mm volume = 353 mm^3^ (B) No plaque detected	(A) 10-degree ventral curvature + 15-degree left curvature (B) None	VAS score = 8 Pain disappeared after 12 months	26 > 27	30 months	Orally: propolis 600 mg + bilberry 160 mg + silymarin 400 mg + ginkgo biloba 250 mg + L-carnitine 1000 mg + coenzyme Q10 100 mg + Boswellia 200 mg + Vitamin E 30 mg/daily/for 30 months + topically: diclofenac gel 4%/2× daily for 30 months Note: The patient refused periplaque penile injections with pentoxifylline
2	52 years	Dupuytren’s disease, Ledderhose disease, congenital dorsal penile curvature (5 degrees).	Middle third + distal third	(A) First plaque: 13.4 × 9.15 × 2.83 mm volume = 182 mm^3^. Second plaque: 14.8 × 8.43 × 3.87 mm. Total volume = 252 mm^3^ (B) No plaque detected	(A) 20-degree dorsal penile curvature + 20-degree left penile curvature (B) 5-degree dorsal penile curvature. Previous condition = congenital dorsal penile curvature (5 degrees)	VAS score = 0	26 > 27	51 months	Orally: propolis 600 mg + bilberry 160 mg + silymarin 400 mg + ginkgo biloba 250 mg + L-carnitine 1000 mg + coenzyme Q10 100 mg + Boswellia 200 mg + vitamin E 30 mg/daily for 51 months + topically: diclofenac gel 4%/2× daily for 51 months + periplaque penile injections: pentoxifylline 100 mg (30 G needle) every 2 weeks for 6 months, and then 1 penile injection every month for 12 months, and 1 penile injection every 2 months for 24 months (42 total injections)
3	30 years	Left varicocele	Proximal third	(A) 12.2 × 11.2 × 2.81 mm volume = 203 mm^3^ (B) No plaque detected	(A) No penile deformation (B) No penile deformation	VAS score = 5 Pain disappeared after 4 months	26 > 26	4 months	Orally: propolis 600 mg + bilberry 160 mg + silymarin 400 mg + ginkgo biloba 250 mg + vitamin E 800 IU/daily for 4 months + topically: diclofenac gel 4%/2× daily for 4 months Note: Penile injections not needed

Abbreviations: IIEF = International Index of Erectile Function; VAS = visual analog pain score.

## Data Availability

Not applicable.
